# TNF Inhibitor Therapy in Corticosteroid‐Resistant or ‐Dependent Pediatric Neutrophilic Dermatosis

**DOI:** 10.1111/pde.70095

**Published:** 2025-11-03

**Authors:** Laure Chêne, Thomas Hubiche, Jean‐Marie De Guillebon, Maella Severino‐Freire, Emmanuelle Bourrat, Sébastien Barbarot, Alice Phan, Christine Chiaverini

**Affiliations:** ^1^ Service de Dermatologie, CHU Nice Nice France; ^2^ Service de Néphrologie, Rhumatologie pédiatrique, Hôpitaux pédiatriques de Nice CHU‐Lenval Nice France; ^3^ Service de Dermatologie CHU Toulouse Toulouse France; ^4^ Service de Dermatologie, Assistance Publique‐Hôpitaux de Paris, Université de Paris Paris France; ^5^ Service de Dermatologie, CHU de Nantes Nantes France; ^6^ Service de Pédiatrie, Hôpital Mère Enfant Bron France

**Keywords:** child, pyoderma gangrenosum, sweet syndrome

## Abstract

Neutrophilic dermatoses are rare in children. Systemic corticosteroids are the first‐line treatment, but guidelines for second‐line therapies are lacking. We report five cases of children with systemic steroid‐resistant/dependent neutrophilic dermatoses, successfully treated with tumor necrosis factor inhibitors.

**Abbreviations**
ND: neutrophilic dermatoses
PG: pyoderma gangrenosum
SS: sweet syndrome
TNFi: TNF inhibitors

## 1 Introduction

Neutrophilic dermatoses (NDs) are characterized by sterile perivascular and diffuse neutrophilic infiltrates in the skin [1, 2]. Pyoderma gangrenosum (PG) and Sweet syndrome (SS) are the two most frequent clinical subtypes. NDs are rare in children with a dermatologic presentation usually resembling that in adults but with a higher frequency of extra‐cutaneous complications [3, 4, 5]. Systemic corticosteroids are the first‐line treatment for NDs. However, clear guidance on second‐line treatment is lacking. Here we report five cases of corticosteroid‐resistant or steroid‐dependent NDs in children treated with tumor necrosis factor (TNF) inhibitors (TNFi).

## 2 Methods

In this retrospective multicenter study, children of both sexes, ≤ 18 years old with a histologically confirmed diagnosis of PG or SS, and non‐response or dependence on systemic corticosteroids (defined by lack of response to a minimal dose of 2 mg/kg/day or relapse of lesions when the dose is decreased) were included after oral consent. A standardized questionnaire was filled using medical records. The study was registered in the treatment registry of Nice University Hospital (reference R04‐049).

## 3 Results

**FIGURE 1:**
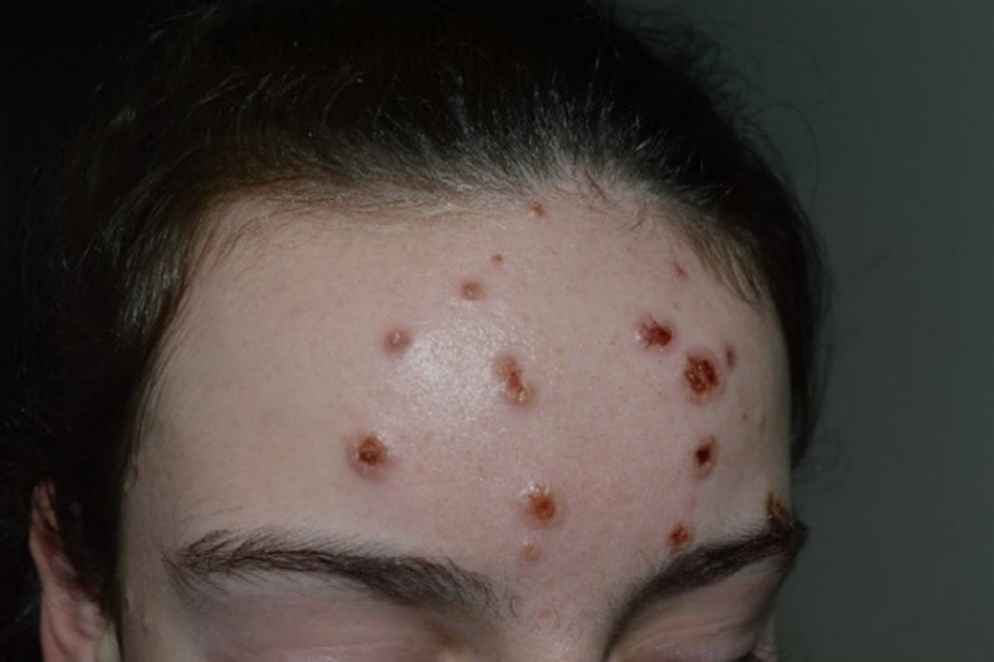
Sweet syndrome of a14‐year‐old child (patient 3) with necrotic papules of the face.

**FIGURE 2:**
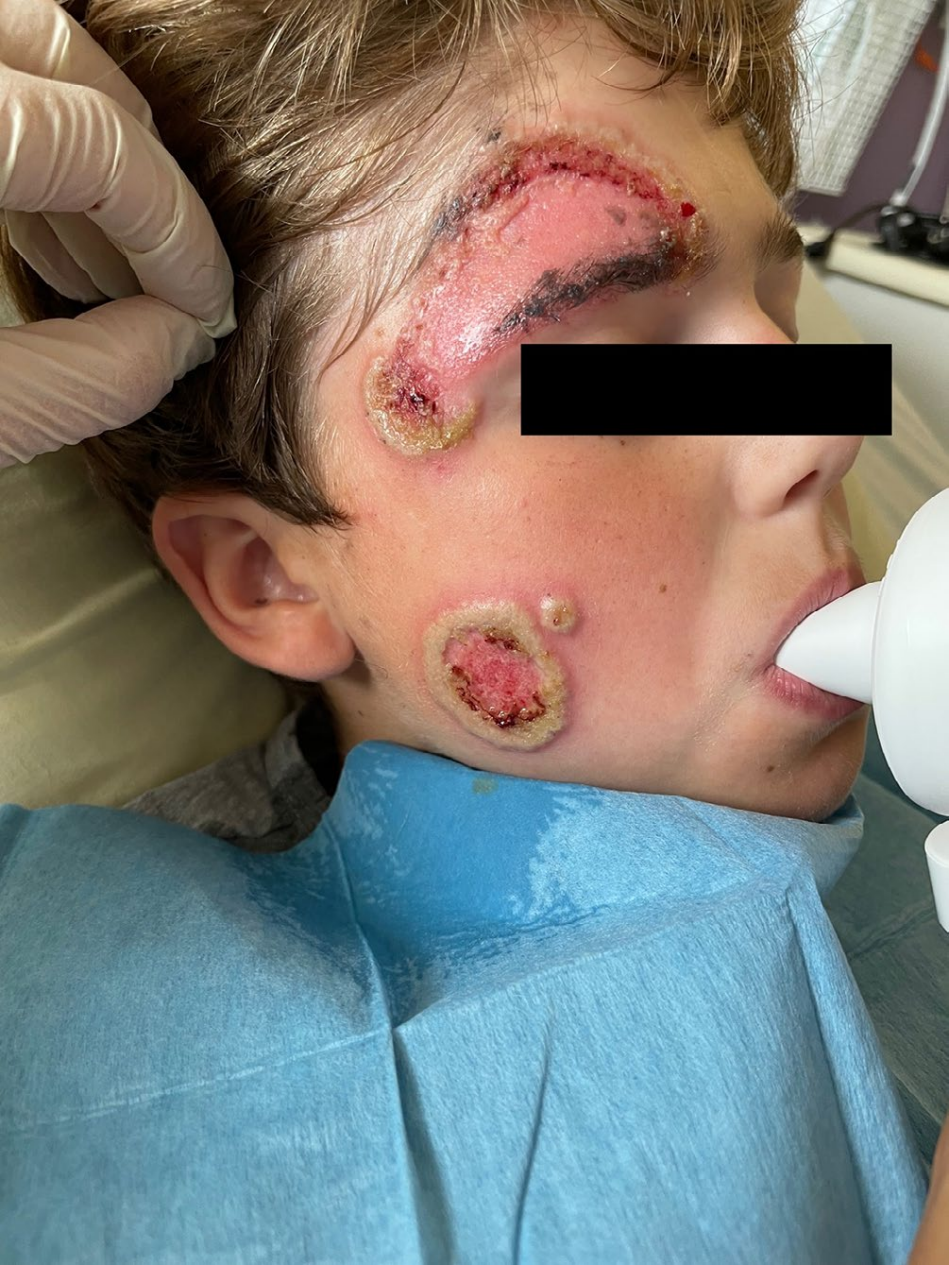
Pyoderma gangrenosum on the face of a 13‐year‐old child (patient 2) with pustular plaques coalesced and evolved into a painful ulcer.

Five children met inclusion criteria for this study: three with PG and two with SS (Table 1). All children had multiple cutaneous painful pustular and/or necrotic nodules without visceral involvement (Figures 1 and 2). Two children had severe mucosal involvement. Associated etiologies included inflammatory bowel disease (IBD) (*n* = 1), familial Mediterranean fever (*n* = 1), airway infections (*n* = 2), BCG vaccination (*n* = 1), and varicella (*n* = 1). All children received oral or intravenous corticosteroids from 2 to 5 mg/kg/day either without efficacy (*n* = 2) or steroid dependence (*n* = 3). Second‐line treatments included dapsone (2 mg/kg/d) without efficacy (*n* = 2), cyclosporine up to 5 mg/kg/day without efficacy (*n* = 2) or complicated by renal toxicity (*n* = 1), and TNFi (*n* = 5). Three children had an infliximab infusion started at different regimens and increased to 10 mg/kg every 2 or 3 weeks with efficacy. In one child, the onset of anti‐infliximab antibodies led to a switch to adalimumab with efficacy. Two children received adalimumab with complete (*n* = 1) or partial (*n* = 1) response. Systemic steroids were stopped in three children after 1–6 months. TNFi were stopped for two children after complete response with limited relapses. Three continued TNFi without skin involvement. No adverse effects were reported.

**TABLE 1:** Patient characteristics.

Patients	Sex/age at onset (years)	Clinical features		Associated disease/event	First line treatment	Second line treatment	Follow‐up adverse event
Patient 1 Sweet syndrome	F/3	Skin	Leg, ankle, arm, head necrotic lesions	Rhinitis/heterozygous *MEFV* gene mutation	*Prednisolone*: 2 mg/kg/day; ineffective, tapered to 0.1 mg/kg/day	*Infliximab*: 10 mg/kg/day every month then every 4 weeks, allowing gradual withdrawal of corticosteroids over 18 months	Recovery but flares triggered by infections No adverse event
		Mucosa	Lesions on the soft palate, anterior lingual border, red lips, base of tongue and aryepiglottic folds, nasal cavity, cavum, left tonsil and supraglottic lesions. Dysphagia and odynophagia.				
		Other	None				
Patient 2 Sweet syndrome	F/14	Skin	Nodular ± purpuric lesions, with hemorrhagic bullae, livedo, necrosis of limbs, face	Streptococcal sore throat	*Prednisolone*: 1.5 mg/kg/day (improvement but dependence and cushingoid face) then stopped after 7 months	*Dapsone*: increased until 100 mg/day (no efficacy) *Cyclosporine*: 4 mg/kg/day (no efficacy) then 5 mg/kg/day: improvement but stopped after 2 years because of nephrotoxicity *Adalimumab*: 40 mg/15 days, then 40 mg/21 days then stopped after 2.5 years without recurrence (5 months)	Recovery No adverse event
		Mucosa	None				
		Other	None				
Patient 3 Pyoderma gangrenosum	M/13	Skin	Pustular and necrotic annular lesions of limbs, face Pathergy+	Inflammatory bowel disease	*Prednisolone*: 1 mg/kg/day then 250 mg IV*3: ineffective then stopped after 1 month	*Cyclosporine*: 4 mg/kg/day: ineffective then stopped after 2 months *Infliximab*: 7.5 mg/kg: initially effective then onset of anti‐infliximab antibodies and resistance to treatment *Adalimumab*: 80 mg then 40 mg/15 days for 1 year	Recovery No adverse event No relapse
		Mucosa	None				
		Other	Inflection of the stature–weight curve and delayed onset of puberty				
Patient 4 Pyoderma gangrenosum	M/6	Skin	Pustular lesions leading to painful ulcerations on the limbs, bottom, and face Pathergy+	BCG vaccination	*Methylprednisolone*: 2 mg/kg/day + oral *Dapsone* (2 mg/kg/day): improvement but persistence of pathergy signs	*Cyclosporine*: 5 mg/kg/day (worsening) *Prednisolone*: up to 5 mg/kg/day then stopped after 6 months + *Infliximab*: 5 mg/kg/14 days then increased at 10 mg/kg/14 days because of laryngitis and respiratory distress, then stopped without recurrence (7 years)	Recovery No adverse event 2 relapses at 3 and 5 months after treatment interruption treated with corticosteroids and colchicine
		Mucosa	Laryngitis				
		Other	Diarrhea and vomiting				
Patient 5 Pyoderma gangrenosum	M/3.5	Skin	Pustular lesions leading to painful ulcerations on limbs and trunk Pathergy+	Varicella	*Prednisolone*: 1 mg/kg/day then 2 mg/kg/day: effective but relapse when decreased, actually 0.5 mg/kg/day	*Adalimumab*: 40 mg then 20 mg/15 days then 20 mg/7 days: improvement	Improvement but flares triggered by infections No adverse event
		Mucosa	None				
		Other	None				

Abbreviations: BCG, Bacillus Calmette–Guérin; MEFV, familial Mediterranean fever.

## 4 Discussion

NDs are rare in children [2]. Severe presentations are possible, with mucosal and upper airway involvement (laryngeal and/or bronchial), as illustrated by two of our cases with respiratory distress risk, or with systemic involvement (not observed in this cohort) [6]. NDs may be idiopathic or associated with chronic inflammatory disease, including IBD, paraneoplastic disorders (including hematologic diseases), immune deficiency, and auto‐inflammatory disorders [7, 8]. In our cohort, four of five cases presented after vaccination or an infectious episode; one had IBD, and another had a heterozygous mutation of *MFEV*, which emphasizes the importance of screening for immunodeficiency and/or auto‐immune inflammatory disorders in this population.

There are no guidelines for management of pediatric ND. As for adults, the first‐line treatment is oral corticosteroids at 0.5–2 mg/kg/day, but some authors reported using corticosteroid pulses from 15 to 30 mg/kg body weight/day for pediatric PG [2]. Recurrence occurs in up to 30%–45%. ND resistant to, or dependent on, systemic corticosteroids is unusual in children [2, 3, 4]. Various second‐line therapies, including cyclosporine have been proposed in the literature with variable efficacy and safety [9]. TNFi seem effective and well‐tolerated in adult patients with ND [10, 11]. In a review of pediatric PG, TNFi, alone or combined with systemic corticosteroids, were used in 23 cases: infliximab 5 mg/kg or adalimumab 40 mg per week with a cure rate of about 60% [9]. Only two children with SS treated with infliximab (6 mg/kg at weeks 0, 2, and 6) have been reported [4, 11]. We found similar results in our patients, with a higher optimal dose of 10 mg/kg every 2 or 3 weeks for infliximab, and 20–40 mg (depending on weight) every 1 or 2 weeks for adalimumab. One limitation of anti‐TNFα drugs is the possible onset of anti‐drug antibodies that can lead to progressive treatment failure. A switch to another TNFi was possible for one of our patients. Efficacy was incomplete for one child. As reported in the literature, the treatments were well‐tolerated in this small cohort, but serious side effects are possible with TNFi. The limitations of this study are a small cohort size and its retrospective and descriptive nature. Larger studies are needed to confirm these data.

## 5 Conclusion

TNFi can be a rapidly effective and safe option for corticosteroid‐resistant or ‐dependent pediatric NDs.

## Author Contributions

**Laure Chêne:** conceptualization, data curation, formal analysis, investigation, methodology, project administration, software, validation, visualization, writing – original draft. **Thomas Hubiche:** writing – review and editing. **Jean‐Marie De Guillebon:** writing – review and editing. **Maella Severino‐Freire:** writing – review and editing. **Emmanuelle Bourrat:** writing – review and editing. **Sébastien Barbarot:** writing – review and editing. **Alice Phan:** writing – review and editing. **Christine Chiaverini:** conceptualization, investigation, methodology, project administration, software, validation, visualization, writing – original draft.

## Conflicts of Interest

The authors declare no conflicts of interest.

## Data Availability

The data that support the findings of this study are available on request from the corresponding author. The data are not publicly available due to privacy or ethical restrictions.
